# Dream Activity in Narcoleptic Patients During the COVID-19 Lockdown in Italy

**DOI:** 10.3389/fpsyg.2021.681569

**Published:** 2021-05-26

**Authors:** Serena Scarpelli, Valentina Alfonsi, Anita D'Anselmo, Maurizio Gorgoni, Alessandro Musetti, Giuseppe Plazzi, Luigi De Gennaro, Christian Franceschini

**Affiliations:** ^1^Department of Psychology, Sapienza University of Rome, Rome, Italy; ^2^IRCCS Fondazione Santa Lucia, Rome, Italy; ^3^Department of Biomedical and Neuromotor Sciences, University of Bologna, Bologna, Italy; ^4^Department of Humanities, Social Sciences and Cultural Industries, University of Parma, Parma, Italy; ^5^IRCCS Istituto delle Scienze Neurologiche di Bologna, Bologna, Italy; ^6^Department of Biomedical, Metabolic and Neural Sciences, University of Modena and Reggio Emilia, Modena, Italy; ^7^Department of Medicine and Surgery, University of Parma, Parma, Italy

**Keywords:** COVID-19 pandemic, creativity, lucid dreaming, nightmares, dream recall, sleep, narcolepsy

## Abstract

Some studies highlighted that patients with narcolepsy type-1 (NT1) experience high lucid dream frequency, and this phenomenon has been associated with a creative personality. Starting from the well-known “pandemic effect” on sleep and dreaming, we presented a picture of dream activity in pharmacologically treated NT1 patients during the Italian lockdown. Forty-three NT1 patients completed a web-survey during Spring 2021 and were compared with 86 matched-controls. Statistical comparisons revealed that: (a) NT1 patients showed greater sleepiness than controls; (b) controls showed higher sleep disturbances than NT1 patients, and this result disappeared when the medication effect in NT1 was controlled; (c) NT1 patients reported higher lucid dream frequency than controls. Focusing on dreaming in NT1 patients, we found that (a) nightmare frequency was correlated with female gender, longer sleep duration, higher intrasleep wakefulness; (b) dream recall, nightmare and lucid dream frequency were positively correlated with sleepiness. Comparisons between low and high NT1 lucid dreamers showed that patients more frequently experiencing lucid dreams reported a greater influence of dreaming during wakefulness, especially concerning problem-solving and creativity. Overall, our results are consistent with previous studies on pandemic dreaming carried out on healthy subjects. Moreover, we confirmed a link between lucidity and creativity in NT1 patients. Considering the small sample size and the cross-sectional design, our findings cannot provide a causal relationship between lucid dreams and the COVID-19 lockdown. Nevertheless, they represent a first contribution to address future studies on this issue, suggesting that some stable characteristics could interact with changes provoked by the pandemic.

## Introduction

Narcolepsy type 1 (NT1) is a neurological disorder characterized by excessive daytime sleepiness and sleep abnormalities, mainly dissociated manifestations of Rapid Eye Movement (REM) sleep. Indeed, rapid access into REM sleep at sleep onset, sleep paralysis, hypnagogic and/or hypnopompic hallucinations, and the pathognomonic symptom cataplexy are key features of this disorder [American Academy of Sleep Medicine (AASM), 2014].

Narcoleptic patients frequently report unusual and emotional dream experiences (Schredl, 1998; Schiappa et al., 2018). Further, the available literature underlined that NT1 patients often experience a scarce insight of what refers to dream content rather than real experience, leading to “delusional confusion” between dreaming and reality (Hays, 1992; Szucs et al., 2003; Wamsley et al., 2014).

Some studies revealed that narcoleptic patients report a more significant level of awareness during sleep mentation than healthy subjects (Lequerica, 1999; Fosse, 2000). More directly, it has been highlighted that narcolepsy is associated with experiencing lucid dreaming (i.e., the phenomenon of being aware of dreaming during dreaming) (Dodet et al., 2015; Rak et al., 2015). Specifically, Rak et al. (2015) carried out an investigation based on phone-interview and showed that 60 narcoleptic patients had higher dream recall frequency, nightmares, and lucid dreams than controls. This finding was supported by an experimental study, revealing that patients had more lucid dreams and were able to indicate their awareness while they were asleep using a defined eye movement signal (Dodet et al., 2015). Other findings underlined a link between the high metacognition during sleep and creativity personality profile (Zink and Pietrowsky, 2013). Interestingly, in narcoleptic patients lucid dreaming is associated with a higher creative profile, and hypnagogic hallucinations lead to higher creative success and potential, impacting on the creative identity of NT1 patients (Lacaux et al., 2019; D'Anselmo et al., 2020).

The latter findings open new perspectives in the investigation of the lucid dreaming process in narcolepsy (Dodet et al., 2015; Rak et al., 2015), and claim for systematic targeted studies comparing healthy and narcoleptic patients.

Further, dream activity and emotional processing are strictly related (Scarpelli et al., 2019a). In this regard, a recent review highlighted that the neurobiological basis of narcolepsy and patients' mental sleep activity appears to be closely associated (Schiappa et al., 2018). Indeed, neuroimaging studies found that narcolepsy is characterized by limbic dysfunction (Poryazova et al., 2009; Meletti et al., 2015; Vaudano et al., 2019). The amygdala and hypothalamus are also involved in processing emotional memory and fear during wakefulness (Richter-Levin, 2004), which may be responsible for disturbing dreams. In this view, dream experiences could be considered the expression of patient well-being or distress and could be clinically relevant.

We have to underline that recent studies highlighted that, during the COVID-19 pandemic, people reported sleep alterations and, in parallel, high dream recall and nightmare frequency (e.g., Iorio et al., 2020; MacKay and DeCicco, 2020; Gorgoni et al., 2021; Scarpelli et al., 2021a). During the pandemic, people's dreams showed a greater negative emotional charge (e.g., Iorio et al., 2020; Schredl and Bulkeley, 2020; Gorgoni et al., 2021; Scarpelli et al., 2021a) and pandemic-related contents (e.g., Iorio et al., 2020; Schredl and Bulkeley, 2020). Moreover, fragmented and lighter sleep has proven to be linked to dreaming during lockdown in healthy subjects (Scarpelli et al., 2021a), consistently to the “arousal-retrieval hypothesis” pointed out that dream recall may be promoted by awakenings during sleep (Koulack and Goodenough, 1976). Further, a gender difference was observed since females revealed a larger dream recall rate according to the previous literature (e.g., Barrett, 2020; Iorio et al., 2020; Scarpelli et al., 2021a). In addition, aging was associated with a drop in dream recall frequency also during lockdown (Scarpelli et al., 2021a).

Here, for the first time, we present a picture of dream activity in pharmacologically treated narcoleptic patients during the pandemic. We intend to provide a better understanding of dreaming in narcolepsy, reporting the results from a cross-sectional study in which we have compared NT1 and healthy subjects. In light of the current background, we expected that NT1 patients had a greater dream activity (dreams, nightmares and lucid dreams) than controls. We also hypothesized that (1) poor sleep quality and high arousal (i.e., frequent awakenings) are associated with dream frequency in NT1 patients; (2) gender and age are related to dream activity during the pandemic in NT1 patients; (3) lucid dream frequency and creativity are linked in NT1 patients, as highlighted by previous literature.

## Materials and Methods

### Study Design, and Participants

Patients who met diagnostic criteria for narcolepsy type 1 (NT1) and followed at the Outpatients Clinical for Narcolepsy at the IRCCS—Institute of the Neurological Sciences of Bologna were requested to fill out a web-survey on the Microsoft Azure platform. Specifically, NT1 subjects had to meet the international criteria for NT1 [American Academy of Sleep Medicine (AASM), 2014].

Also, the web-survey was promoted by means of university communication systems and virtual learning environments to recruit healthy subjects. The survey lasted about 30 min and was addressed to over-18 exclusively.

All questionnaires were collected during spring 2020. Specifically, the survey was available online from March 10, 2020, to May 4, 2020.

Firstly, subjects filled out a self-administered questionnaire on socio-demographic information. In addition, psychological measures, sleep measures and dream variables were collected by specific self-reported questionnaires.

All subjects signed an electronic informed consent before accessing the questionnaires. The subjects also explicitly agreed to provide an email contact and created an identification code to anonymize it. The study was conducted in accordance with the Declaration of Helsinki and was approved by the local ethic committee (Comitato Etico Indipendente di Area Vasta Emilia Centro—CE-AVEC).

Overall, 60 NT1 patients received the link to fill out the web-survey. A total of 43 NT1 patients completed the survey (response rate: 71.67%). In keeping with many studies including patients with rare conditions (e.g., Fortuyn et al., 2010) we chose to compare NT1 patients and healthy controls with a ratio case/matched controls of 1:2 to increase statistical power. Hence, 86 healthy controls were extracted from respondents to the web-survey (Franceschini et al., 2020b) and were matched for age, gender, Italian area, and days elapsed since the beginning of lockdown.

All NT1 patients were on pharmacological treatment: 28 patients on pharmacotherapy with Sodium oxybate; 15 patients on pharmacotherapy with other medications (e.g., Pitolisant; Modafinil). Data from healthy subjects reported in the current study have been presented elsewhere (Franceschini et al., 2020b; Scarpelli et al., 2021a) and were part of a wider project, “Resilience and the COVID-19: how to react to perceived stress. Effects on sleep quality and diurnal behavior/thoughts,” with different objectives concerning the impact of lockdown in the Italian population.

### Measures

The webform was composed of 4 sections:

#### Socio-Demographic and COVID-19-Related Information

This section allowed us to collect the following variables: age, gender, marital status, presence/absence of children, education level, Italian area, occupation, forced quarantine, having COVID-19-infected friends or relatives.

#### Psychological Measures

Psychological measures were evaluated by the Italian version of the Depression Anxiety Stress Scale (DASS-21) short form (Bottesi et al., 2015), a self-administered questionnaire in which subjects rate the frequency and severity of depression, anxiety, and stress symptoms. The 21 Items refer to the previous week and each item is scored on a 4-point scale (0 = “Did not apply to me at all,” to 3 = “Applied to me very much, or most of the time”). Anxiety, depression and stress scores result from the sum of the responses to the items from each subscale multiplied by 2 to suit the original 42 items. The cut-offs for severe rating of depression, anxiety and stress are ≥21, ≥15, and ≥26, respectively (Lovibond and Lovibond, 1995).

#### Sleep Measures

Sleep measures were assessed by the Italian adaptation of the Medical Outcomes Study- sleep scale (MOS-SS) (Palagini and Manni, 2016), a self-reported questionnaire including 12 items to assess sleep quality and quantity within a 4-week period. Ten of the 12 MOS-SS items are scored on a 6-point categorical scale ranging from “1 = all of the time” to “6 = none of the time.” The question about the time required to fall asleep uses a 5-point categorical response scale ranging from “0 to 15 min,” to “more than 60 min.” “Sleep duration” is reported by subjects as the average number of hours they sleep each night. All domains except sleep duration are converted from 0 to 100, and item 2 is recorded as the average number of hours slept per night (0–24 h). This instrument provides six measures for sleep quality: sleep disturbance, snoring, awakening short of breath or with headache, sleep adequacy, somnolence and sleep duration/optimal sleep. Finally, MOS-SS has a global index to assess quality of sleep defined Sleep Index II or Sleep problem index, an aggregate measure of responses to nine of the questions about the four sleep domains (sleep disturbance, awakening short of breath or with headache, sleep adequacy and sleepiness). Scores for the MOS-SS Sleep Index II range from 0 to 100, with higher scores indicating greater sleep problems (cut-off = 25.8; Hays et al., 2005). In keeping with previous work (Scarpelli et al., 2021a), we have taken into consideration also self-reported evaluation of the intrasleep wakefulness (item 8), dichotomized as follows: “high intrasleep wakefulness” (answer “3,” “4,” “5”) and “low intrasleep wakefulness” (answer “1,” “2”).

#### Dream Variables

Dreaming was assessed by the Italian adaptation of the Mannheim Dream Questionnaire (MADRE) (Settineri et al., 2019), a self-reported questionnaire with 20 items. Dream recall frequency (item 1) was rated by a 7-point scale format (0 = never and 6 = almost every morning). Nightmare frequency (item 4) and lucid dream frequency (item 10) was rated by an 8-point scale (0 = never and 8 = several times a week). The dream and nightmare frequency were asked with reference to the last month. Also, emotional intensity, emotional tone and nightmare distress were evaluated (item 2, 3, 5). Individuals' attitudes toward dreams (i.e., the personal meaning of one's own dreams and the impression that dreams provide impulses or pointers for waking life) were assessed by item 12, consisting of eight sentences with a 5-point format (0 = Not at all/4 = Totally). The impact of dream on daily life (the frequency of dream sharing, the recording of dreams, the dreams affecting day-time mood, the creative. dreams, and the problem-solving dreams, from item 13 to 17). Dream variables eliciting utilizations of dreams (i.e., frequency of dream sharing, recording of dreams, dreams affecting day-time mood, creative dreams, problem-solving dreams) were in 8-point scale with 0 = never and 8 = several times a week.

Further, the questionnaire required information on presence/absence and percentage of recurring nightmares (item 6, 7) and on and deja-vu experiences based on dream (item 18). Additionally, the questionnaire includes specific items on age of first lucid dream (item 11); reading about dreams (item 19, rated using a 3-points scale) and helpful dream literature (item 20, rated using a 5-points scale).

### Statistical Analysis

Descriptive analyses were conducted to outline the sociodemographic characteristics of the sample, considering the following features: age, gender, marital status, presence/absence of children. education level, occupation, forced quarantine, having COVID-19-infected friends or relatives.

Firstly, the Mann-Whitney U tests were used to compare differences between the NT1 and control group concerning the psychological measures extracted from DASS (anxiety, depression and stress) and sleep variables extracted from MOS-SS (sleep disturbances, snoring, awakening short of breath or with headache, sleep adequacy, sleepiness, and sleep problem index) and the oneiric measures regarding dream frequency (dreams, nightmares, lucid dreams) and qualitative emotional features (emotional intensity, emotional tone and nightmares distress).

We performed non-parametric Spearman's correlations (rank bivariate or rank biserial, depending on the variable type) to assess the relationship between NT1 dreaming (dream recall frequency, nightmare frequency, lucid dream frequency) and (1) demographic factors (age, gender) previously associated to oneiric activity (Scarpelli et al., 2021a) and (2) sleep measures (sleep duration and intrasleep awakenings extracted from MOS-SS, sleep disturbances, snoring, sleepiness) to test the arousal-retrieval hypothesis.

In order to better understand the relationship between lucid dreaming and the waking-correlates of oneiric activity in narcolepsy, we carried out statistical comparisons between high and low lucid dreamers (Mann-Whitney U test), considering the following independent variables: attitude toward dreams (items 12—averaged score), and items regarding utilization of dreaming during wakefulness (item 13-frequency of dream sharing, item 14-recording of dreams, item 15- dreams affecting day-time mood, item 16- creative dreams, item 17- problem-solving dreams). Item 10 about lucid dreaming was dichotomized, as follows: “low lucid dreamers” (answer from 0-never to 5-about 2/3 times a month; *N* = 27) and “high lucid dreamers” (answer from 6-about once a week to 7-several times a week; *N* = 16).

The statistical analyses were performed using Statistical Package for Social Sciences (SPSS) version 25.0 and Matlab R2019a. *P*-values of < 0.05 were considered statistically significant.

## Results

### Characteristics of Samples

The characteristics of participants are shown in Table 1. In short, 37.2% of the samples were young subjects (age 18–25). Female gender was represented by 58.1% of the samples. Subjects were mainly single, both in NT1 (37.2%) and HC group (36%). Further, most of the participants received high school education in NT1 (55.8%) and HC group (33.7%). A high percentage of respondents were employed both in NT1 (55.8%) and HC group (58.1%). Further, 58.1% came from the North of Italy in both groups. Also, more than 70% of the individuals of each sample did not have children. The majority of the respondents from both groups did not experience a forced quarantine (97.7 %). Among healthy individuals, 14% had COVID-19-infected friends or relatives, while in the NT1 group only a subject had a significant other infected.

**Table 1:** Socio-demographic and COVID-19-related characteristics.

	**Controls (*N* = 86) N (%)**	**Narcoleptic patients (*N* = 43) N (%)**
**Age**		
18–25	32 (37.2)	16 (37.2)
26–30	10 (11.6)	5 (11.6)
31–40	20 (23.3)	8 (18.6)
41–50	20 (23.3)	12 (27.9)
51–60	4 (4.7)	2 (4.7)
**Gender**		
Male	36 (41.9)	18 (41.9)
Female	50 (58.1)	25 (58.1)
**Marital status**		
Single	31 (36)	16 (37.2)
Married	22 (25.6)	7 (16.3)
Cohabitating	12 (14)	10 (23.3)
Engaged	20 (23.3)	8 (18.6)
Divorced/separated/widower	1 (1.2)	2 (4.7)
**Education level**		
Until middle School	1 (1.2)	4 (9.3)
High school	29 (33.7)	24 (55.8)
Bachelor's degree	25 (29.1)	8 (18.6)
Master's degree	24 (27.9)	4 (9.3)
PhD/postgraduate school	7 (8.1)	3 (7)
**Occupation**		
Student	29 (33.7)	14 (32.6)
Employed	50 (58.1)	24 (55.8)
Retired	1 (1.2)	0 (0)
Unemployed	6 (7)	5 (11.6)
**Italian area**		
North Italy	50 (58.1)	25 (58.1)
Center and South Italy	36 (41.9)	18 (41.9)
**Having children**		
Yes	25 (29.1)	11 (25.6)
No	61 (70.9)	32 (74.4)
**Forced quarantine**		
Yes	2 (2.3)	1 (2.3)
No	84 (97.7)	42 (97.7)
**COVID-19-infected friends/relatives**		
Yes	12 (14)	1 (2.3)
No	74 (86)	42 (97.7)

### Comparisons Between Narcoleptics and Controls

Table 2 summarized the statistical comparisons between the NT1 and control group, considering psychological, sleep and dream measures. The results revealed no difference in psychological measures. Conversely, concerning sleep variables, NT1 patients reported significantly higher sleepiness than controls (*p* < 0.001). Also, healthy individuals reported higher sleep complaints than NT1 patients (*p* < 0.001). Considering that the NT1 group was on pharmacological treatment, we performed a control analysis to check whether the pharmacotherapy could impact self-reported sleep disturbances among patients (please, see the Supplementary Material). This supplementary analysis showed that the difference in sleep disturbances between controls and NT1 subjects is maintained only when patients were treated with Sodium oxybate, a pharmacological treatment promoting slow-wave sleep and sleep quality (Franceschini et al., 2020a). Conversely, the difference in sleep disturbances disappears comparing controls with NT1 patients treated with other types of medications.

**Table 2:** Mean rank of psychological, sleep and dream measures and results of statistical comparisons (Mann-Whitney U Test) between controls and narcoleptic patients.

		**Mean rank**	**U-values**	**Z-scores**	***p-values***
Anxiety	C	63.74	1740.5	−0.549	0.483
	NT1	67.52			
Depression	C	66.44	1,725	−0.633	0.225
	NT1	62.12			
Stress	C	67.81	1,607	−1.212	0.534
	NT1	59.37			
Sleep disturbances	C	73.08	1,154	−3.485	** <0.001**
	NT1	48.84			
Snoring	C	62.55	1,638	−1.222	0.222
	NT1	69.91			
Awakenings (short of breath or headache)	C	65.69	1,790	−0.371	0.710
	NT1	63.63			
Sleep adequacy	C	60.58	1,469	−1.914	0.056
	NT1	73.84			
Sleepiness	C	53.01	818	−5.200	** <0.001**
	NT1	88.98			
Sleep problem index	C	67.26	1,655	−0.971	0.332
	NT1	60.49			
Dream recall frequency	C	63.21	1,695	−0.789	0.430
	NT1	68.58			
Nightmares frequency	C	65.58	1,799	−0.253	0.800
	NT1	63.84			
Lucid dream frequency	C	59.36	13,643	−2.449	**0.014**
	NT1	76.28			
Emotional intensity	C	65.97	1,766	−0.434	0.664
	NT1	63.07			
Emotional tone	C	64.06	1,768	−0.435	0.663
	NT1	66.88			
Nightmares distress	C	62.31	1,618	−1.191	0.234
	NT1	70.37			

*α = 0.05. Values in bold indicate significant difference*.

*C, Controls; NT1, narcoleptic patients type 1*.

Crucially, patients showed higher lucid dreams than controls (*p* = 0.014). No other significant difference was found regarding dream frequency and dream emotionality.

### Demographic and Sleep Features Related to Dream Frequency

Spearman's correlation analyses (see Table 3) showed that (a) nightmare frequency in the NT1 group was related to female gender (*p* = 0.030), longer sleep duration (*p* = 0.043), higher intrasleep wakefulness (*p* = 0.006); (b) dream recall frequency, nightmare frequency and lucid dream frequency were positively correlated with sleepiness (*p* = 0.003).

**Table 3:** Features related to dream frequency in narcoleptic patients: Spearman's correlation coefficients.

	**Dream recall frequency**		**Nightmare frequency**		**Lucid dream frequency**	
	**Correlation**	**p-values**	**Correlation**	**p-values**	**Correlation**	**p-values**
Age	−0.116	0.457	−0.067	0.667	−0.174	0.264
Gender	0.079	0.612	0.331	**0.030**	0.010	0.951
Sleep duration^a^	0.123	0.461	0.331	**0.043**	0.016	0.924
Intrasleep wakefulness	0.138	0.379	0.411	**0.006**	0.138	0.379
Sleep disturbances	0.110	0.481	0.258	0.095	0.069	0.661
Snoring	−0.11	0.942	0.024	0.877	0.016	0.918
Sleepiness	0.443	**0.003**	0.465	**0.002**	0.391	**0.009**

a *Correlations performed on 38 narcoleptic patients for missing data on this item*.

*α = 0.05. Values in bold indicate significant difference*.

### Comparisons Between High and Low Lucid Dreamers

Table 4 summarized the statistical comparisons between low and high NT1 lucid dreamers revealing that high lucid dreamers, as compared to low lucid dreamers: (a) had higher mean score in the factor “attitude toward dream” (*p* = 0.010); (b) more frequently recorded their dreams (*p* = 0.036); (c) more frequently had dreams influencing their creativity during wakefulness (*p* = 0.011); (d) more frequently had dreams influencing their problem-solving during wakefulness (*p* = 0.001).

**Table 4:** Mean rank of attitude toward dreams (averaged score), items regarding utilization of dream during wakefulness and results of statistical comparisons (Mann-Whitney U Test) between narcoleptic patients with low and high lucid dreamers.

		**Mean rank**	**U-values**	**Z-scores**	**p-values**
Attitude toward dreams	LLD	18.22	114	−2.568	**0.010**
	HLD	28.38			
Dream sharing	LLD	21.52	203	−0.330	0.741
	HLD	22.81			
Recording of dreams	LLD	20.02	162.5	−2.092	**0.036**
	HLD	25.34			
Dreams affecting day-time mood	LLD	19.61	151.5	−1.652	0.099
	HLD	26.03			
Creative dreams	LLD	18.37	118	−2.544	**0.011**
	HLD	28.13			
Problem-solving dreams	LLD	17.52	95	−3.242	**0.001**
	HLD	29.56			

*α = 0.05*.

*LLD, low lucid dreamers; HLD, high lucid dreamers*.

*Values in bold indicate significant difference*.

## Discussion

For the first time, the current study aimed to investigate pandemic's dream activity in NT1 patients. Overall, we revealed that NT1 patients showed greater sleepiness than healthy subjects, consistently to their diagnosis [American Academy of Sleep Medicine (AASM), 2014]. In contrast, the healthy group reported higher sleep disturbances than NT1 patients as measured by MOS-SS. This result could appear surprising, nevertheless, we showed that the difference disappeared when matched-controls were compared with NT1 patients treated with medications different from Sodium oxybate. In other words, the observed difference considering the whole NT1 group can be ascribed to effective treatment with Sodium oxybate on nocturnal sleep, which reduced disturbed sleep across the night (Franceschini et al., 2020a). In parallel, it should be noted that our data were collected during Spring 2020. In this regard, several studies demonstrated that the pandemic is provoking remarkable sleep alterations in healthy subjects (Blume et al., 2020; Casagrande et al., 2020; Cellini et al., 2020; Franceschini et al., 2020b; Wright et al., 2020), especially causing insomnia symptoms (i.e., COVID-Somnia; Gupta and Pandi-Perumal, 2020).

However, studies that investigated nocturnal sleep quality in narcoleptic patients during lockdown reported mixed results. Changes in sleep quality resulted related to changes in work habits in adult NT1 patients in the study of Postiglione et al. (2021). They found no significant alterations in patients working or studying at home and increased sleep disturbances in patients that do not change their working schedule (Postiglione et al., 2021). A study on NT1 and NT2 patients reported an increase in sleep fragmentation in 43.4% and not change in 35.5% of the sample (Rodrigues Aguilar et al., 2020). Finally, recent data on pharmacologically treated NT1 children and adolescents reported no differences in sleep quality during lockdown compared to an earlier period (Filardi et al., submitted).

Concerning the primary objective of our investigation, we revealed that only lucid dreams frequency significantly differed between groups. In line with previous literature (Dodet et al., 2015; Rak et al., 2015), patients with narcolepsy had higher lucid dreams frequency than healthy subjects. Surprisingly, no difference was found in emotional dream features.

Consistently to the findings on healthy subjects during lockdown (e.g., Scarpelli et al., 2021a), we found that nightmares in NT1 patients were more frequent in women. This result is also in line with the evidence that females reported higher rates of negative emotions in their sleep mentation during pandemic (Barrett, 2020). Barrett suggests that this gender difference may be explained in light of the “continuity-hypothesis,” considering women more vulnerable to anxiety and mood alteration than men during lockdown (Barrett, 2020). The impact of gender on dream activity is well-established (Schredl and Reinhard, 2008) and may be independent by the COVID-19 health emergency.

It should be noted that the cross-sectional protocol makes it difficult to draw definitive conclusions about the link between pandemic and the differences highlighted between NT1 patients and control subjects. Moreover, the small sample size of NT1 patients and a response rate of around 70% could represent a serious weakness of our study. On the one hand, the limited number of patients involving in the study is not surprising since NT1 is a rare condition affecting 0.02–0.06% of adults, as highlighted from epidemiological studies in the United States and Europe (e.g., Mignot et al., 1997; Longstreth et al., 2007; Kornum et al., 2017). On the other hand, we have to mention that the small NT1 sample size did not allow us to study directly the impact of adverse events (forced quarantine, COVID-19-infected relatives or friends) on emotional dream features since the pandemic-related conditions were reported in ~2% of our sample. In fact, all patients and healthy controls spent that period in conditions similar to quarantine due to the lockdown from March 10, 2020, to May 4, 2020, that forced people to stay at home in confinement. For all these reasons, our considerations should be taken with caution. Actually, we can only describe a picture of dream activity in narcolepsy during Spring 2020 without disentangling whether we observed a stable phenomenon in NT1 or a condition influenced by the COVID-19 emergency (i.e., stay-at-home in confinement).

Although we showed that sleep disturbances were attenuated by pharmacotherapy in the NT1 group, we revealed that sleepiness was correlated with dream recall, nightmare, and lucid dream frequency. In keeping with the activation-hypothesis (D'Atri et al., 2019; Scarpelli et al., 2021b) and the so-called arousal-retrieval model (Koulack and Goodenough, 1976), this result may be interpreted as an index of lighter nocturnal sleep. More directly, a greater amount of intrasleep wakefulness was positively associated with nightmare frequency, as highlighted by previous evidence (Scarpelli et al., 2021a). Awakenings during sleep would guarantee the elaboration of dream material and its transfer from short-term memory storage to long-term storage (Koulack and Goodenough, 1976; Scarpelli et al., 2021b).

Interestingly, NT1 patients, as well as control subjects (see Scarpelli et al., 2021a) reported an association between frightening dreams and higher sleep duration. This finding is only apparently in conflict with the activation/arousal-retrieval model. In fact, the phenomenon of sleep extension during lockdown has been described by Bottary et al. (2020) highlighting that even if people spontaneously prolonged their sleep duration, the rest could be featured by poor quality and fragmentation, and confirmed by recent data (Alfonsi et al., 2021).

The comparisons between high and low lucid dreamers confirmed that the increased awareness of their own mental sleep activity was linked to the perception that the oneiric dimension has a remarkable impact on daily-life. Specifically, the greater problem-solving and creative dreams in high lucid dreamers are consistent with the studies suggesting that lucid dreaming and dissociated REM sleep manifestations are associated with creativity in narcoleptic patients (Lacaux et al., 2019; D'Anselmo et al., 2020). Additionally, it is worth noting that a greater awareness of dreams may have a role in emotional regulation (Scarpelli et al., 2019a). Again, it should be mentioned that our results cannot provide a direct association between pandemic, creativity and lucid dreams in NT1 patients. However, in keeping with the available literature (Dresler et al., 2014; Rak et al., 2015; Schiappa et al., 2018), we can speculate that narcoleptic patients with more frequent experiences of lucid dreams have developed a coping strategy in their dream experience to face the negative emotional contents linked to the COVID-19 emergency. In this view, narcoleptic patients reporting high lucid dreams felt that lucidity provides relief for their nightmares (Rak et al., 2015). We have also to consider that high lucid dreamers in our sample reported a greater attitude toward oneiric experience, being more prone to record their dreams. In other words, these subjects reported high dream salience (Cohen, 1979) that may be considered an intraindividual stable and COVID-unrelated feature affecting dream recall frequency (Schredl and Göritz, 2015).

## Conclusions

The main finding of the current study is the presence of higher lucid dreams frequency in NT1 patients than healthy subjects during pandemic. In line with previous studies, we highlighted that sleep measures influenced oneiric activity (Bottary et al., 2020; Gorgoni et al., 2021; Scarpelli et al., 2021a) and female gender was linked with higher nightmare frequency (Barrett, 2020; Scarpelli et al., 2021a). In addition, NT1 high lucid dreamers had more dream salience and their oneiric activity appeared to significantly affect the wakefulness (Schiappa et al., 2018). Interestingly, we confirmed a link between lucid dreaming and the creative dimension in NT1 patients.

Although we reported for the first time a picture of dreaming and sleep pattern in NT1 patients during pandemic, we have to emphasize that our findings cannot provide a causal relationship between lucid dreams and the COVID-19 lockdown. Only a longitudinal design could clarify whether the changes detected are linked to the pandemic or whether they are stable features in the investigated groups. Indeed, using retrospective questionnaires represents an important limitation of our study. In this regard, the literature points to that perspective diaries and collecting dream reports immediately after awakening are more reliable methods to obtain information on dreaming (Scarpelli et al., 2019b). Similarly, an intrinsic limitation of the study concerning the use of a self-reported questionnaire to assess sleep patterns without the support of any objective measures (e.g., actigraphy).

Finally, we believe that the present study offers a first contribution to address future research on COVID-19 outbreak and individual's well-being, suggesting that some trait-like characteristics such as individual attitude toward dreams, gender and also underlying sleep disorders could interact with changes provoked by the pandemic.

## Data Availability Statement

The raw data supporting the conclusions of this article will be made available by the authors, without undue reservation.

## Ethics Statement

This study was conducted in accordance with the Declaration of Helsinki and the study protocol was approved by the local ethics committee (Comitato Etico Indipendente di Area Vasta Emilia Centro—CE-AVEC). Electronic informed consent was obtained from each participant prior to starting the investigation. Participants could withdraw from the survey at any moment without providing any justification. The patients/participants provided their written informed consent to participate in this study.

## Author Contributions

SS and VA provided substantial contributions to the conception of the work, deep analysis of the literature, study design, development, and final approval of the manuscript. CF and LD contributed in the design of the study and participated in the development and revision of the work and agreement for final approval of the manuscript. AD'A and MG contributed to data analysis and agreement for final approval of the manuscript. AM and GP contributed to the revision of the work and agreement for final approval of the manuscript. GP, LD, and CF contributed to deep revision of the work, with literature analysis and agreement for final approval of the manuscript contributed to deep revision of the work, with literature analysis and agreement for final approval of the manuscript. All authors contributed to the article and approved the submitted version.

## Conflict of Interest

GP is a consultant and participated in advisory board for UCB Pharma, Jazz pharmaceuticals, Bioprojet, Takeda, and Idorsia outside the submitted work. The remaining authors declare that the research was conducted in the absence of any commercial or financial relationships that could be construed as a potential conflict of interest.
