# Supplementary material for: Dream Activity in Narcoleptic Patients During the COVID-19 Lockdown in Italy
Source: Front Psychol. 2021 May 26;12:681569. doi: 10.3389/fpsyg.2021.681569 (PMC8187856; doi:10.3389/fpsyg.2021.681569)
Supplement: Supplementary file 1 [file Data_Sheet_1.docx]

**Supplementary materials**

**Control analysis on sleep disturbances**

Considering that the narcoleptic patients type 1 (NT1) were on pharmacological treatment, we performed a control analysis to check whether the pharmacotherapy could impact self-reported sleep disturbances among patients. Firstly, we divided the NT1 groups in patients treated with Sodium oxybate (28 subjects) and patients treated with other medications (15 subjects).

The Mann-Whitney U tests were then used to compare differences concerning “sleep disturbances” (from MOS-SS) between each NT1 subgroup and their matched-control group. The following tables S1 and S2 summarize the results (*controls vs. NT1 subgroup*).

**Table S1. Controls vs. NT1 subgroup (other medication)**

|  | ***Mean Rank*** | ***U*** | ***Z*** | ***p*** |
| --- | --- | --- | --- | --- |
| **Matched-control group**  **N=30** | 24.58 | 177.5 | -1.147 | 0.251 |
| **NT1 – other medication group**  **N=15** | 19.83 |  |  |  |

**Table S2. Controls vs. NT1 subgroup (sodium oxybate)**

|  | ***Mean Rank*** | ***U*** | ***Z*** | ***p*** |
| --- | --- | --- | --- | --- |
| **Matched-control group**  **N=56** | 48.80 | 431 | -3.368 | **0.001** |
| **NT1 – sodium oxybate group**  **N=28** | 29.89 |  |  |  |

This supplementary analysis showed that sleep disturbances between controls and NT1 subjects are maintained only when patients were treated with Sodium oxybate (p=0.001), a pharmacological treatment promoting sleep continuity and deep sleep (Franceschini et al., 2020). Conversely, the difference on sleep disturbances disappears comparing controls with NT1 patients treated with other type of medications (p=0.251).

**References**

Franceschini, C., Pizza, F., Antelmi, E. et al. Narcolepsy treatment: pharmacological and behavioral strategies in adults and children. Sleep Breath 24, 615–627 (2020). https://doi.org/10.1007/s11325-019-01894-4
